# Development and Validation of Earthquake Fire Response Simulation Protocol for Korean College Students in Health Programs

**DOI:** 10.3390/ijerph19095764

**Published:** 2022-05-09

**Authors:** Hyun-Ok Jung, Seung-Woo Han

**Affiliations:** 1 College of Nursing, Kyungpook National University, Daegu 41944, Korea; juiris@korea.kr; 2 Department of Emergency Medical Technology, Kyungil University, Gamasilgil Hayangeup, Gyeongsan 38428, Korea

**Keywords:** disaster safety, earthquake response, fire response, evacuation, fire suppression, disaster response capacity

## Abstract

The purpose of this study is to evaluate the adequacy of the developed protocol by verifying the validity of the expert group for the earthquake and fire response simulation protocol. A protocol development team consisting of one emergency rescue professor, one counseling psychology professor, three paramedics, and one firefighter developed the study’s protocols to promote the core response and capabilities required at an earthquake fire site. We checked the content validity for the appropriateness of the contextual connection for each stage for the protocol. We also created an evaluation checklist to measure the items for each stage. The protocol developed in this study consists of earthquake response, fire response, evacuation, and fire suppression. We set the situation for each stage and composed learner activities and learning performance goals. The earthquake response stage included (1) shout “it’s an earthquake,” (2) protect yourself, (3) turn off electricity and gas, and (4) evacuate to a safe place. In the fire response stage, (1) shout “fire,” (2) press the emergency bell and call 119, (3) close the door of a dangerous place where fire can spread, and (4) evacuate to a safe place. In the evacuation stage, (1) open the emergency exit, (2) cover your nose and mouth, (3) lower your posture, and (4) evacuate quickly in one direction. Lastly, in the firefighting stage, (1) pull out the safety pin, (2) hold the nozzle and face the fire, (3) grab the handle, and (4) spray the powder evenly. The protocol contributes to the development of systematic and elaborate simulation education materials in the future. Furthermore, it provides basic data for future disaster simulation operation and protocol development through continuous training and practical exercises.

## 1. Introduction

In recent years, natural disasters have continued worldwide. Among them, earthquakes are unpredictable and unexpected, moving away from certain areas affected by the circum-Pacific orogenic zone [1,2]. In particular, in Korea, the 5.4 Richter magnitude earthquake in Pohang in 2017 was the second largest after the Gyeongju earthquake in 2016 since the Korea Meteorological Administration started earthquake observation. The earthquake’s damage was even more significant because its epicenter was close to the downtown population and buildings [3]. Significantly, damage in a densely populated city center can threaten people’s lives and safety and create substantial economic loss [4]. In addition, a previous study [5] mentioned fire as the most dangerous thing after an earthquake, and the more extensive the range of fire after the earthquake, the more time and financial burden for recovery. Therefore, we should recognize earthquakes and fires as a series of disaster events rather than separate events. However, people tend to hesitate in disasters and fail to concentrate on dangerous situations [6]. In previous studies, people who experienced disasters, such as earthquakes, were more likely to have psychological instability, including anxiety and depression. To cope properly without hesitation, disaster education and training are necessary [6,7].

In Korea, awareness of disaster safety is increasing day by day. The Ministry of Public Safety and Security designates mandatory education on disaster safety for employees working at public health centers and fire stations every year. However, there are many limitations in enhancing disaster preparedness capacity. For example, the Ministry only holds one annual ‘Multiple Casualty First Aid Training’ and ‘Comprehensive Emergency Rescue Training for Disaster Preparedness’ [8]. In addition, there must be continuity of disaster safety education to help promote an appropriate response in a disaster. Therefore, the purpose of this study is to evaluate the adequacy of the developed protocol by verifying the validity of the expert group for the earthquake and fire response simulation protocol. We intend to contribute to the development of systematic and elaborate simulation education materials in the future. Recently, simulation education for college students in healthcare has been increasing daily in Korea, and disaster education and training are essential for responding to and preventing disasters [9]. In particular, simulation education in nursing effectively promotes quick decision-making and problem-solving ability and acquires and applies various cases in the field [10]. This research provides a protocol to cope with earthquake fires, most commonly in disasters. Therefore, the protocol developed in this study is intended to provide basic data for correctly performing step-by-step response stage in the event of earthquake and fire for Korean College Students in Health Programs and to utilize this protocol in various occupational groups in the future.

## 2. Materials and Methods

### 2.1. Research Design

This study is a methodological study to evaluate the adequacy of the developed protocol by verifying the validity of the contents of the expert group for the earthquake and fire response simulation protocol.

### 2.2. Study Subjects and Protocol Composition

**Figure 1:**
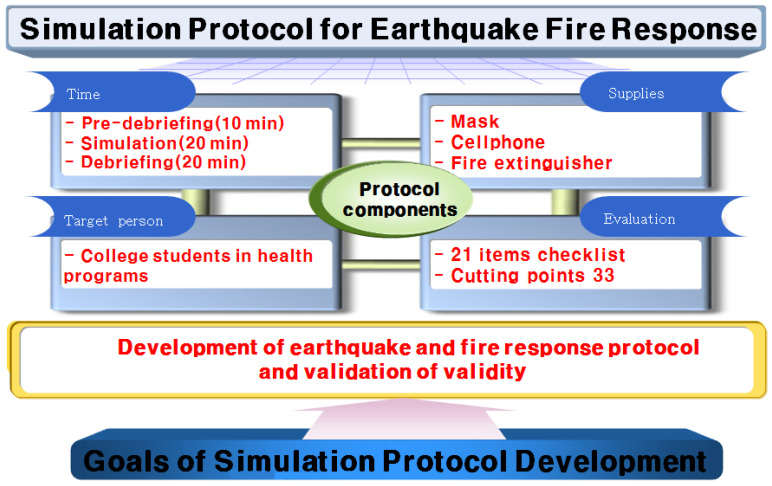
Development of simulation protocol for earthquake fire response.

The theme of this study is ‘Development and Validation of Earthquake Fire Response Simulation Protocol’. We recruited a protocol development team consisting of one emergency rescue professor, one counseling psychology professor, three paramedics, and one firefighter to evaluate the adequacy of the protocol content structure. The subjects of this study were students majoring in Health Programs. The simulation training in a team unit was composed of a group of five people. In the simulation operation method, this protocol takes about 20 min and consists of four stages: earthquake response, fire response, evacuation, and fire suppression. A protocol was constructed in which the simulation was run for about 20 min until the participant achieved the educational goal of each step. If the participants did not reach the educational goal after 20 min, the simulation protocol was set to end. The simulation process consisted of five students in the practice class suddenly feeling vibration, detecting objects shaking severely, and then recognizing an earthquake and hiding under a desk. At the same time, the protocol includes a fire that occurs due to an electric leak, causing an emergency. At this time, students should quickly evacuate to a safe place while protecting themselves, perform proper fire response, and implement fire suppression. We set out to prepare masks, telephones, and fire extinguishers to facilitate earthquake and fire response in this protocol. The preparation time consisted of 10 min of pre-briefing, 20 min of simulation, and 20 min of debriefing (Figure 1).

### 2.3. Protocol Content Adequacy Evaluation and Validity

Four experts verified the protocol developed in this study, including one firefighter, one industrial health manager, one hospital emergency responder, and one first aid instructor. In addition, this protocol included four steps referencing the Crisis Training Guideline [11] of the Ministry of Public Safety and Security and the National Disaster Safety Portal [12]. We checked the content validity for appropriate evaluation items according to the flow of each execution stage and the appropriateness of the contextual connection for each step. At each stage, we supplemented and corrected other opinions from experts. This checklist was composed of 21 items, total 42 points, out of ‘2 points’ if the student performed each item, and ‘0 points’ if not performed. Moreover, in this study, with the advice of four experts, we created an evaluation checklist for practical application of the protocol (Table 1). Finally, the final revised and supplemented checklist was assigned to 16 field experts such as emergency medical technology, nursing, and firefighting to evaluate the appropriateness of the contents.

The content expert evaluation calculated the mean and standard deviation, and the validity of each item was confirmed using the CVR (Content Validity Ratio) proposed by Lawshe [13] to evaluate the content validity of each item. In addition, a previous study [14] presented the criteria for content validity evaluation, and it was said that there was no problem with item content adequacy if the number of panels was 15 or more and the CVR value was 0.49 or more.

### 2.4. Ethical Approval

The purpose and process of the study were explained to 16 field experts, and they agreed to participate in the study voluntarily, and it was explained that they would not be disadvantaged by giving up halfway. In addition, the researcher directly explained that all matters recorded are computerized anonymously, so personal secrets are guaranteed. It was investigated anonymously for confidentiality and collected in sealed envelopes. K University’s IRB committee confirmed that it was not a study that actually applied or harmed the subjects due to the classification of the study, and it was confirmed that it would not require ethical approval. Therefore, it was excluded from IRB deliberation.

## 3. Results

The 16 experts who evaluated the relevance of the content filled out a questionnaire composed of 21 items on a Likert scale ranging from 0 points of ‘not at all appropriate’ to 4 points of ‘very appropriate’.

The relevance of the contents of the expert’s checklist presented in this study ranged from the highest point of 4.00 (±0.00) to the lowest point of 3.25 (±1.29). The CVR values ranged from 0.500 to 1.000 and all exceeded the standard value of 0.49, confirming that there was no abnormality in content validity (Table 1). After expert consensus, the evaluation score cutting point was 33 out of 42. Looking at the general characteristics of the 16 experts, the gender was the same with 8 males and females. Participants in their 50s formed the most numerous group, and the 30s and 40s showed the same ratio. As for the level of education, doctorate degrees were the most common, and the main form of employment was emergency medical technology. The years of service were at a similar rate for less than 5 years and between 5 and 10 years, and the simulation teaching experience was high in ‘yes’. Both disaster and fire response education experiences and certificates showed high ‘yes’ and ‘have’ (Table 2).

**Figure 2:**
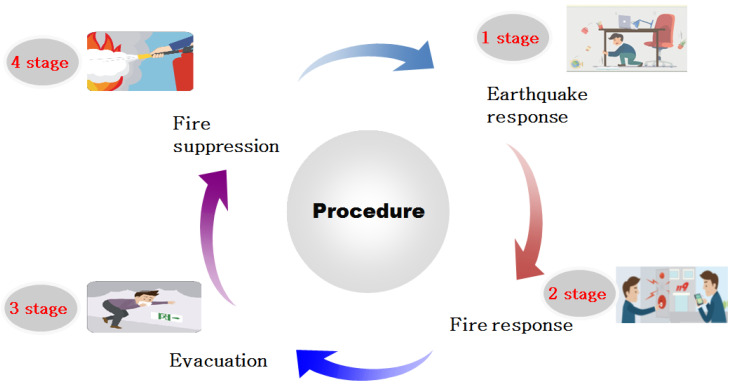
Simulation protocol procedure.

The final protocol developed in this study is an earthquake fire response simulation protocol, consisting of seismic response, fire response, evacuation, and fire suppression. Each protocol stage included a situation setting, learner activity, and learning performance goal (Figure 2). The first stage in this protocol is to respond to earthquakes, and consists of four steps: The student who senses an earthquake must (1) shout, “It’s an earthquake”, (2) evacuate themselves to a safe place, (3) turn off electricity and gas when the shaking stops, and (4) secure an exit and evacuate quickly. These four steps provide appropriate actions in responding to earthquakes in the learning performance goal. The second stage in this protocol is when a fire caused by a wire short circuit occurs after an earthquake in a classroom. The second stage has four steps: The student witnessing the fire must (1) shout, ‘Fire’, (2) ring the emergency bell and call 119, (3) close the door in a dangerous place (which may spread the fire), and (4) secure an exit and evacuate as soon as possible. The learning performance goal in fire occurrence is to perform the initial response, report after a fire actively, and evacuate quickly. The third stage in this protocol is evacuation. It consists of four steps: (1) open the emergency exit and close the door in the dangerous place, (2) cover your nose and mouth with a handkerchief or clothes, (3) take a lower position than the smoke, and (4) hold the wall with one hand and evacuate quickly in one direction. In the learning performance goal, these four steps calmly reassure the subject, secure a safe distance, and engage the learner to perform emergency evacuation actions appropriately. Finally, the fourth stage in this protocol is fire suppression. Like the other stages, it also consists of four steps: (1) pull out the fire extinguisher safety pin, (2) point the fire extinguisher hose towards the fire, (3) grasp the handle, and (4) shoot towards the fire. Through these four steps, it is possible to perform fire suppression properly and correctly operate the fire extinguisher in the learning performance goal (Table 3).

## 4. Discussion

This protocol consists of four stages (earthquake response, fire response, evacuation, and fire suppression). Within each stage are four steps for appropriate action measures and crisis response.

In the earthquake response stage, these protocol settings require you to DROP where you are to your hands and knees before the earthquake knocks you down to keep you safe during an earthquake. Then, you should be able to cover your head, neck, and possibly your entire body under a sturdy desk or table. The context is consistent with the prior study [15] of performing HOLD ON to your shell (or your head and neck) until the shaking stops. In this study protocol, we emphasized the importance of shouting on detecting an earthquake and quickly trapping your body inside heavy objects.

We also highlighted the importance of emphasizing the initial response through simulation to cut off electricity and gas to reduce fire occurrence. Through these considerations, we believe that learners can complete the earthquake response protocol successfully. In this protocol, we set not only earthquake response but also fire response. Since the existing earthquake simulation emphasizes only earthquake response methods, it is a limitation that simulation settings accompanied by fire are rare. In a prior study [16], researchers mentioned the importance of early extinguishment because fire accompanying earthquakes would make it difficult to extinguish and directly increase damage to buildings. Therefore, in our study’s protocol, four steps settings are in a similar context to those in another study [17] that highlighted the fire procedure of R.A.C.E (Rescue. Alert. Confine. Extinguish.) before extinguishing the fire. However, this study included a step emphasizing reporting and evacuation rather than rescue because the subjects were students in health programs. Therefore, we set more realistic expectations for these participants, including using professional skills rather than rescue skills.

In this study’s protocol, there are also four items in the third step. This protocol set is in line with the part mentioned in the previous study [18], where researchers stated that you should stay as far away from the danger zone as possible and crawl if possible. Therefore, this protocol emphasized covering the nose and mouth in case of fire and moving quickly with the lowest position possible. This protocol means that even if someone knows about the crisis, they may be nervous and slow to cope with the situation, so training is necessary to master accurate and straightforward skills. Moreover, this protocol’s steps highlight the importance of emergency evacuation for immediate use. In addition, we accentuate the continuous practice and familiarization of the four steps so that the students can understand easily. Finally, in the fire suppression stage, we set up four steps for correct fire extinguisher use. This protocol setting is consistent with a previous study’s [19] stress on P.A.S.S (Pull. Aim. Squeeze. Sweep.). This protocol also focused on creating a friendly environment to easily follow the steps for fire extinguisher use, as emphasized in previous studies. In four stages and four steps in each stage, this study’s protocol will contribute to developing health-related college students’ capacities for managing earthquake fire emergencies. Continuous training and practical exercises based on these protocols provide essential data for future disaster simulation operations. Considering the previous simulations and training experiences for earthquake and fire response, most public institutions viewed earthquakes and fires separately. It was very rare for the general public to approach earthquakes and fires simultaneously except for special situations such as the training of firefighters. Therefore, the protocol developed in this study is meaningful in that it operates simulations that apply earthquake and fire to health-related college students simultaneously, providing basic data. Thus, based on this study, it is expected that the simulation protocol developed through expert verification can be used as an educational program for other occupational groups or population groups.

This study developed a protocol for applying the simulation of earthquake and fire response to health-related college students. First, because this protocol is for college students in health-related programs, the study’s limitation is that it did not cover all age groups. Therefore, researchers should develop protocols for the elderly and children. Second, as this study focused on the simulation of an actual earthquake and fire response, it did not deal with the psychological first aid part that helps individuals cope in disasters. As a result, future research will also need to develop education and protocol on psychological first aid that should accompany earthquake fires. Lastly, since disasters are sudden and unpredictable, it is considered to be very important to develop a protocol taking into account the disaster response ability, awareness, weather, and situation of the subject. In the future, it is hoped that the simulation protocol will be developed and applied by subdividing it into beginner, intermediate, and advanced courses in the future in consideration of students’ disaster response ability, awareness, weather, and building situation.

## 5. Conclusions

This study developed an earthquake fire response simulation protocol and evaluated the protocol content suitability through expert evaluation. Our study had a total of four stages (earthquake response, fire response, evacuation, fire suppression) and four steps in each stage. In this study, the validity of the simulation protocol was verified by 16 experts, and the CVR (Content Validity Ratio) value was found to exceed the reference point of 0.49 in all items, confirming that there was no problem in applying the protocol.

The four-stage four-step model developed in this study includes simple, clear, and easy-to-follow protocols and contributes to providing basic data for the development of various earthquake disaster simulations.

## Figures and Tables

**Table 1:** Evaluation checklist.

Performance Step	Evaluation Item		Evaluation Score			Expert Evaluation of Content		
			Performed	Lacking	Not Performed	M	SD	CVR
Preparation	1	Check site safety	2	1	0	3.50	1.12	0.500
	2	Hand hygiene (use hand sanitizer)	2	1	0	3.25	1.29	
	3	Read the protocol and introduce your role	2	1	0	3.63	0.50	
Earthquake response	4	(Earthquake situation occurs) React to an earthquake by shouting, ‘It’s an earthquake’.	2	1	0	4.00	0.00	0.875
	5	(Desks or hard objects provided) Protect yourself to keep as safe as possible	2	1	0	4.00	0.00	
	6	(When the shaking stops) Turn off electricity and gas	2	1	0	3.81	0.75	
	7	(After securing the exit) Quickly evacuate to a safe place	2	1	0	3.88	0.34	
Fire response	8	(Witnesses of a fire) Responds to the fire by shouting, “Fire!”	2	1	0	4.00	0.00	0.875
	9	Press the emergency bell and call 119	2	1	0	4.00	0.00	
	10	Close the doors of dangerous places where fire can spread	2	1	0	3.75	0.58	
	11	(After securing the exit) Quickly evacuate to a safe place	2	1	0	4.00	0.00	
Evacuation	12	Open emergency exit	2	1	0	3.81	0.54	0.750
	13	Cover your nose and mouth with a handkerchief or clothing	2	1	0	3.94	0.25	
	14	Take a lower stance than smoke	2	1	0	3.88	0.34	
	15	Evacuate quickly in one direction while placing your hand against the wall	2	1	0	3.75	0.58	
Fire suppression	16	(Wear a mask) Pull out the fire extinguisher safety pin	2	1	0	3.94	0.25	1.000
	17	Point the fire extinguisher hose towards the fire	2	1	0	3.88	0.34	
	18	Grab the handle	2	1	0	3.88	0.34	
	19	Shoot it to the fire (End it when the supervisor gives instructions to end)	2	1	0	3.94	0.25	
Finish	20	Perform hand hygiene	2	1	0	3.25	1.00	0.500
	21	Participant carries out the protocol safely and accurately and identifies and resolves the problem within the given time	2	1	0	3.75	0.58	
Evaluation Score Cutting Point After Expert Agreement			33 out of 42 points					

**Table 2:** General characteristics of experts.

Variable	Classification	Expert Proportion	
		N	%
Gender	Man	8	50.0
	Woman	8	50.0
Age	30–39	5	31.3
	40–49	5	31.3
	50–59	6	37.4
Education level	Master’s degree	1	6.3
	Completed the PhD course	2	12.5
	Doctoral degree	13	81.2
Employment major	Nursing	5	31.3
	Emergncy Medical Technology	7	43.8
	Fire safety	3	18.8
	Other	1	6.1
Years of service (yr)	Less than 5 years	7	43.8
	More than 5 years and less than 10 years	6	37.5
	More than 10 years	3	18.7
Simulation-related teaching experience	Yes	12	75.0
	No	4	25.0
Educational experience related to disaster and fire response	Yes	12	75.0
	No	4	25.0
Qualifications related to disaster and fire response	Have	7	43.8
	None	9	56.2

**Table 3:** Simulation protocol for earthquake fire response.

Performance Step-Time (Minutes)	Learner Activity	Learning Performance Goal
1. Earthquake response (4 min)	Students who sense an earthquake shout, ‘It’s an earthquake!’	- After an earthquake, learners can perform the initial action actively.
	A disaster warning siren sounded, and the subject carries out earthquake actions.	
	- All students hide under their desks.	- Able to perform earthquake actions.
	- When the shaking stops, shut off the nearest gas and electricity as soon as possible.	- Possible to prevent the occurrence of additional risks accompanying an earthquake.
	- Evacuate as soon as possible after securing an exit.	- Secure a safe distance and evacuate quickly.
2. Fire response (4 min)	At the same time as an earthquake happened, a fire occurred due to an electric leakage.	
	- Students who sense a fire shout, ‘Fire!’ three times.	- After a fire, learners can carry out the initial reaction actively.
	- When the emergency bell activated and the fire alarm siren sounded, students become aware of the fire situation.	- Learners can perform the fire evacuation alarms properly.
	- Report 119 using a mobile phone (inform of the current fire situation, such as time, location, and expected number of victims). - Notify students that they will take action according to the instructions of the 119 phone call.	- Actively carry out the reporting procedure and the protocol.
	- Close the door in a dangerous place that could cause a fire.	- Quickly perform blocking.
	- Secure an exit and evacuate immediately.	- Secure a safe distance and evacuate quickly.
3. Evacuation (6 min)	- Open emergency exits and close doors in dangerous places, and notify the place to gather.	- Learners can check and properly guide emergency evacuation on each floor (information about prohibiting the use of elevators and closing fire doors in case of evacuation).
	- Cover your nose with your clothes or handkerchief and take a lower position than the smoke. - Quickly evacuate in one direction while placing your hand against the wall. - Move as far away as possible from the fire.	- Learners can carry out evacuation while calmly reassuring the subject and securing a safe distance. - Able to actively carry out actions according to evacuation.
4. Fire Fighting (6 min)	- Evacuate students quickly and attempt to extinguish the fire.	- Learners can carry out fire suppression properly after they evacuate the students to a safe place based on each action step (secure fire extinguishers and wear masks).
	- Initial fire extinguishing with a fire extinguisher. (Position toward the fire with the provided fire extinguisher without spraying) ① Pull out the fire extinguisher safety pin. ② Point the fire extinguisher hose towards the fire. ③ Grab the handle. ④ Shoot it around the flames like sweeping with a broom.	- Can perform appropriate fire extinguisher operation.
End	- The simulation ends when fire suppression completes.	- Simulation finalization and cleanup.

## Data Availability

Data is contained within the article.
